# Serum proteomic identification and validation of two novel atherosclerotic aortic aneurysm biomarkers, profilin 1 and complement factor D

**DOI:** 10.1186/s12953-023-00212-x

**Published:** 2023-08-05

**Authors:** Yusuke Murakami, Mitsuhiro Nishigori, Hiroaki Yagi, Tsukasa Osaki, Masaki Wakabayashi, Manabu Shirai, Cheol Son, Yutaka Iba, Kenji Minatoya, Kengo Kusano, Tsutomu Tomita, Hatsue Ishibashi-Ueda, Hitoshi Matsuda, Naoto Minamino

**Affiliations:** 1 Fundamental Research Laboratory, Research and Development Division, Eiken Chemical Co., Ltd., 143 Nogi, Nogimachi, Shimotsuga-gun, Tochigi, 329-0114 Japan; 2 Omics Research Center, National Cerebral and Cardiovascular Center, 6-1 Kishibe-Shimmachi, Suita, Osaka 564-8565 Japan; 3 Department of Molecular Pharmacology, National Cerebral and Cardiovascular Center Research Institute, 6-1 Kishibe-Shimmachi, Suita, Osaka 564-8565 Japan; 4 Department of Chemistry, Faculty of Science, Fukuoka University, 8-19-1 Nanakuma, Jonan-ku, Fukuoka, 814-0180 Japan; 5 FCM Business Development, HUP Business, Sysmex Corporation, 1-6-23 Goinoikemachi, Nagata-ku, Kobe, 653-0851 Japan; 6 Department of Biochemistry and Molecular Biology, Graduate School of Medical Science, Yamagata University, 2-2-2 Iidanishi, Yamagata, 990-9585 Japan; 7 Department of Diabetes and Endocrinology, Kobe City Nishi-Kobe Medical Center, 5-7-1 Kojidai, Nishi-ku, Kobe, 651-2273 Japan; 8 Department of Vascular Surgery, National Cerebral and Cardiovascular Center, 6-1 Kishibe-Shimmachi, Suita, Osaka 564-8565 Japan; 9 Department Cardiovascular Surgery, Sapporo Medical University School of Medicine, 291 Nishi 16-chome Minami 1-jo, Chuo-ku, Sapporo, 060-8543 Japan; 10 Department Cardiovascular Surgery, Kyoto University Graduate School of Medicine, 54 Shogoin-Kawahara-cho, Sakyo-ku, Kyoto, 606-8507 Japan; 11 Department of Cardiovascular Medicine, National Cerebral and Cardiovascular Center, 6-1 Kishibe-Shimmachi, Suita, Osaka 564-8565 Japan; 12 National Cerebral and Cardiovascular Center Biobank, National Cerebral and Cardiovascular Center, 6-1 Kishibe-Shimmachi, Suita, Osaka 564-8565 Japan; 13 Department of Pathology, National Cerebral and Cardiovascular Center, 6-1 Kishibe-Shimmachi, Suita, Osaka 564-8565 Japan; 14 Present address: Department of Biochemistry, National Cerebral and Cardiovascular Center Research Institute, 6-1 Kishibe-Shimmachi, Suita, Osaka 564-8565 Japan

**Keywords:** Aortic aneurysm, Biomarker, Proteome analysis, Blood test, Discovery, Validation

## Abstract

**Background:**

Effective diagnostic biomarkers for aortic aneurysm (AA) that are detectable in blood tests are required because early detection and rupture risk assessment of AA can provide insights into medical therapy and preventive treatments. However, known biomarkers for AA lack specificity and reliability for clinical diagnosis.

**Methods:**

We performed proteome analysis of serum samples from patients with atherosclerotic thoracic AA (TAA) and healthy control (HC) subjects to identify diagnostic biomarkers for AA. Serum samples were separated into low-density lipoprotein, high-density lipoprotein, and protein fractions, and the major proteins were depleted. From the proteins identified in the three fractions, we narrowed down biomarker candidates to proteins uniformly altered in all fractions between patients with TAA and HC subjects and evaluated their capability to discriminate patients with TAA and those with abdominal AA (AAA) from HC subjects using receiver operating characteristic (ROC) analysis. For the clinical validation, serum concentrations of biomarker candidates were measured in patients with TAA and AAA registered in the biobank of the same institute, and their capability for the diagnosis was evaluated.

**Results:**

Profilin 1 (PFN1) and complement factor D (CFD) showed the most contrasting profiles in all three fractions between patients with TAA and HC subjects and were selected as biomarker candidates. The PFN1 concentration decreased, whereas the CFD concentration increased in the sera of patients with TAA and AAA when compared with those of HC subjects. The ROC analysis showed that these proteins could discriminate patients with TAA and AAA from HC subjects. In the validation study, these candidates showed significant concentration differences between patients with TAA or AAA and controls. PFN1 and CFD showed sufficient area under the curve (AUC) in the ROC analysis, and their combination further increased the AUC. The serum concentrations of PFN1 and CFD also showed significant differences between patients with aortic dissection and controls in the validation study.

**Conclusion:**

PFN1 and CFD are potential diagnostic biomarkers for TAA and AAA and measurable in blood samples; their diagnostic performance can be augmented by their combination. These biomarkers may facilitate the development of diagnostic systems to identify patients with AA.

**Supplementary Information:**

The online version contains supplementary material available at 10.1186/s12953-023-00212-x.

## Background

Aortic aneurysm (AA) is mainly caused by remodelling of the aortic wall by atherosclerosis and leads to disastrous consequences of aortic dissections and ruptures [1–5]. As AA progresses without symptoms in most cases, initial detection often occurs by chance during clinical imaging examinations, such as ultrasound or computed tomography, for other suspected diseases or during voluntary health examinations [6]. Through early detection and rupture risk assessment of AA, patients can increase opportunities for appropriate medical therapy and preventive treatment [7–10]. Thus, the development of sensitive and specific diagnostic tests for detecting AA is urgently needed. Although efforts have been made, the diagnostic biomarkers that are measurable in blood samples have not yet been identified, except for C-reactive protein (CRP) and D-dimer with low specificity and reliability [11, 12]. Omics analysis is considered a rational approach for this purpose; however, available omics data on AA are limited owing to low availability, high heterogeneity, and RNA instability of the AA tissue [13, 14].

Using proteome analysis of aortic tissue from patients with atherosclerotic thoracic AA (TAA) and healthy control (HC) subjects, we recently identified two novel biomarkers of AA that are detectable in blood samples: Niemann-Pick disease type C2 protein (NPC2) and insulin-like growth factor-binding protein 7 (IGFBP7) [15]. To establish a clinical diagnostic system to identify patients with AA with high precision, more biomarker candidates that reflect various aspects of pathogenesis and pathophysiology of AA are warranted. However, in the previous search for biomarkers using aortic tissue proteome analysis, most proteins that elicited dynamic changes in the TAA tissue were excluded, primarily owing to a lack of alteration in their blood level. As there is no such gap in the identification of biomarkers using blood proteome analysis, we decided to search for novel biomarkers by the proteome analysis method using serum samples from patients with atherosclerotic TAA. Nevertheless, this approach is challenging as the high concentrations of abundant proteins, such as albumin, prevent the detection of low-concentration proteins and their alterations [16–18]. In the discovery study, we adopted the lipoprotein sequential flotation ultracentrifugation (SFUC) method and the major protein-depletion method for the pre-treatment of blood samples for proteome analysis to address the above-mentioned challenge.

The fractions obtained were digested and subjected to shotgun proteome analysis. From the identified proteins, profilin 1 (PFN1) and complement factor D (CFD) were selected as novel biomarker candidates for AA, and significant changes in their serum concentration were confirmed in patients with TAA and abdominal (AAA) when compared with those in the HC subjects. In the validation study, we used blood samples and clinical data stored in the biobank of our institute and measured biomarker concentrations in patients with atherosclerotic TAA and AAA and control subjects. PFN1 and CFD showed significant differences in their serum concentrations between patients with TAA or AAA and control subjects. Using the receiver operating characteristic (ROC) analysis, these biomarkers were verified to have sufficient diagnostic power to discriminate patients with TAA and AAA from control subjects. We also measured the serum levels of PFN1 and CFD in patients with aortic dissection (AD) in the validation study to investigate their applications in other related diseases.

## Methods

### Serum sample collection for proteome analysis in the discovery study

Blood samples of patients with TAA and AAA pathologically diagnosed with atherosclerotic AA and whose aortic tissues were used for the identification of NPC2 and IGFBP7 were sampled at the National Cerebral and Cardiovascular Center (NCVC) immediately prior to implantation surgery for blood vessel prosthesis, whereas blood samples of HC subjects were collected at Eiken Chemical Co., Ltd. (ECC) during health examinations. The HC subjects were selected among the volunteers based on the criteria that the biochemical test measures were within the normal limits and no symptoms of cardiovascular and respiratory diseases were present. The characteristics of the enrolled participants are presented in Additional File 1. Blood samples were maintained at 25 °C for 30 min in the presence of a coagulation accelerator and then centrifuged at 1,500 × *g* for 15 min. The resulting serum samples were aliquoted and stored at -80 °C before use.

### Serum sample collection of patients with AA and related diseases in the validation study

Serum samples of patients with TAA, AAA, AD, and inherited arrhythmia (IA) stored in the NCVC Biobank were used for the validation study. We enrolled IA patients with no history of AA, atherosclerotic disease, hyperlipidaemia, hypertension, or diabetes as control subjects because the NCVC Biobank did not collect blood samples from HC subjects. Among patients with TAA, AAA and AD, those with hereditary connective tissue diseases such as Marfan and Ehlers-Danlos syndrome, those with renal insufficiency and familial hypercholesterolemia, or those who underwent aortic surgery before blood sample collection were excluded. In all these cases, patients with two or more AAs and ADs were excluded. TAA and AAA of the patients enrolled in the validation study were considered to be atherosclerotic based on laboratory tests and diagnostic imaging, although not all of them were pathologically diagnosed.

### Serum fractionation using lipoprotein SFUC for serum proteome analysis

The low- and ultralow-density lipoprotein (LDL), high-density lipoprotein (HDL), and protein fractions were prepared using the lipoprotein SFUC method [19]. Three pooled serum samples (TAA1, TAA2, and TAA3) from each of the four patients with TAA (0.075 mL/person) and one pooled serum sample (HC) each from 12 HC subjects (0.025 mL/person) were collected in separate tubes, and the density of each sample was adjusted to 1.063 g/mL by adding 0.7 mL of 0.72 M KBr solution containing 0.75 M NaCl. The serum samples were centrifuged on an ultracentrifuge with a fixed-angle rotor at 40,000 rpm for 20 h at 16 °C. After ultracentrifugation, the upper layer (0.5 mL) was transferred to a new tube to obtain the LDL fraction. Next, 0.7 mL of 3.0 M KBr solution containing 2.6 M NaCl was added to the lower layer (0.5 mL) of each sample and mixed to adjust its density to 1.21 g/mL, and the samples were ultracentrifuged at 40,000 rpm for 48 h at 16 °C. The upper layer (0.6 mL) was transferred to a new tube as the HDL fraction, whereas the remaining lower layer was transferred to another tube as the protein fraction. Highly abundant proteins were depleted from the protein fraction using a Seppro IgY14 LC2 column (Sigma-Aldrich, St. Louis, MO, USA). Finally, the HDL, LDL, and protein fractions (designated as SFUC fractions in total) were subjected to repeated dilution and centrifugal ultrafiltration, to attain a final volume of 0.3 mL with reduced salt concentrations. Protein concentration was measured using a Qubit fluorometer (Life Technologies, Carlsbad, CA, USA). The obtained samples were stored at -80 °C until proteome analysis.

### Liquid chromatography-tandem mass spectrometric (LC–MS/MS) analysis and protein identification

Samples for mass spectrometric (MS) analysis were prepared according to our previous reports, with some modifications [15, 20]. The three SFUC fractions (200 μg protein for each fraction) of TAA1, TAA2, TAA3, and HC samples were individually mixed with sodium deoxycholate (final concentration: 0.5%) and heated at 95 °C for 10 min to denature proteins and inactivate intrinsic proteases. Each fraction was then reduced and alkylated with the FOCUS Protein Reduction-Alkylation Kit (Geno Technology, St. Louis, MO, USA), according to the manufacturer’s instructions. The alkylated samples were diluted fivefold with 50 mM ammonium bicarbonate and digested with lysyl endopeptidase (2 μg; FUJIFILM WAKO Pure Chemicals, Osaka, Japan) at 37 °C for 3 h, and then with trypsin (4 μg; sequence grade; Promega, Madison, WI, USA) at 37 °C for 20 h. The deoxycholate was removed using the phase-transfer method [21], and the resulting digests were desalted using C18 Stage Tips [22]. Tryptic peptides were dissolved in 20 μL of 0.1% formic acid, and their concentrations were measured with a 5-µL aliquot using a Qubit fluorometer. Tryptic peptides (500 ng) were separated using an Eksigent Ekspert NanoLC 425 system (AB SCIEX, Framingham, MA, USA), with a Nano cHiPLC column (75 μm × 150 mm, ChromXP C18-CL, 3 μm, 120 Å; AB SCIEX), and a gradient elution of acetonitrile in 0.1% formic acid from 2.0 to 24.5% for 120 min. The column effluent was introduced into a TripleTOF 5600 tandem mass spectrometer (AB SCIEX) and subjected to high-resolution MS analysis, and the top 10 precursor peptide ions were selected for the subsequent MS/MS analysis. The mass ranges were set at *m/z* 400–1,250 for MS scan and *m/z* 100–1,600 for the MS/MS scan. Peptides were identified using the MASCOT software (version 2.6.1; Matrix Science, London, UK) and Swiss-Prot as the reference database. An MS/MS ion search was performed with a peptide tolerance of 20 ppm and MS/MS tolerance of 0.05 Da. The number of allowed missed cleavages of trypsin digestion was up to one. Pyroglutamination of the N-terminal glutamine residues and methionine oxidation were set as variable modifications. The unique peptides identified with an expectation value of *p* < 0.05 were considered reliable. Nano-LC–MS and MS/MS analyses of tryptic peptides of each sample were performed in duplicate, and proteins identified in both analyses were selected, and are listed in Additional File 2.

### Western blot analysis

After 20-fold dilution, unfractionated serum samples (0.5 μL-equivalents) from individuals were subjected to SDS–polyacrylamide gel electrophoresis in a 10–20% gradient gel (ATTO, Tokyo, Japan) and transferred onto a polyvinylidene difluoride membrane. After blocking, the membrane was incubated with the primary antibodies: mouse monoclonal anti-human profilin 1 antibody (MAB7779, R&D Systems, Minneapolis, MN, USA) or goat polyclonal anti-human CFD antibody (AF1824, R&D Systems). After washing with phosphate-buffered saline containing 0.05% Tween 20, the membrane was incubated with the secondary antibodies: horseradish peroxidase-labelled anti-mouse immunoglobulin G (#7076, Cell Signalling Technology, Danvers, MA, USA) or TidyBlot western blot detection reagent (STAR209P, Bio-Rad, Hercules, CA, USA). Protein bands were visualised using an ECL Prime kit (GE Healthcare), detected using an LAS-2000 Image Analyser (Fujifilm, Tokyo, Japan), and quantified using the ImageJ software v1.53a (Rasband, W.S., NIH, Bethesda, MD, USA).

### Enzyme-linked immunosorbent assay (ELISA)

The concentrations of serum PFN1 and CFD were measured using the human profilin 1 ELISA Kit (CUSABIO, Houston, TX, USA) and human complement factor D Quantikine ELISA Kit (R&D Systems), respectively, according to the manufacturer’s protocols.

### Statistical analysis

BellCurve for Excel (Social Survey Research Information, Tokyo, Japan) and GraphPad Prism software (GraphPad Software, San Diego CA, USA) were used for statistical analyses. The Mann–Whitney *U* test was used to compare two groups and the Steel–Dwass test was used to evaluate the three groups. In these analyses, results with *p* < 0.05 were considered significant. When ROC analysis was performed, the area under the curve (AUC) was calculated to evaluate the capability of biomarker candidates to discriminate patients with atherosclerotic AA (TAA or AAA) from controls (HC subjects or control IA patients). To evaluate the combined AUCs for the two protein biomarkers, a discriminant analysis was performed, and the obtained discriminant score was subjected to ROC analysis.

## Results

### Proteome analysis of fractionated serum samples from patients with TAA and HC subjects

**Fig. 1:**
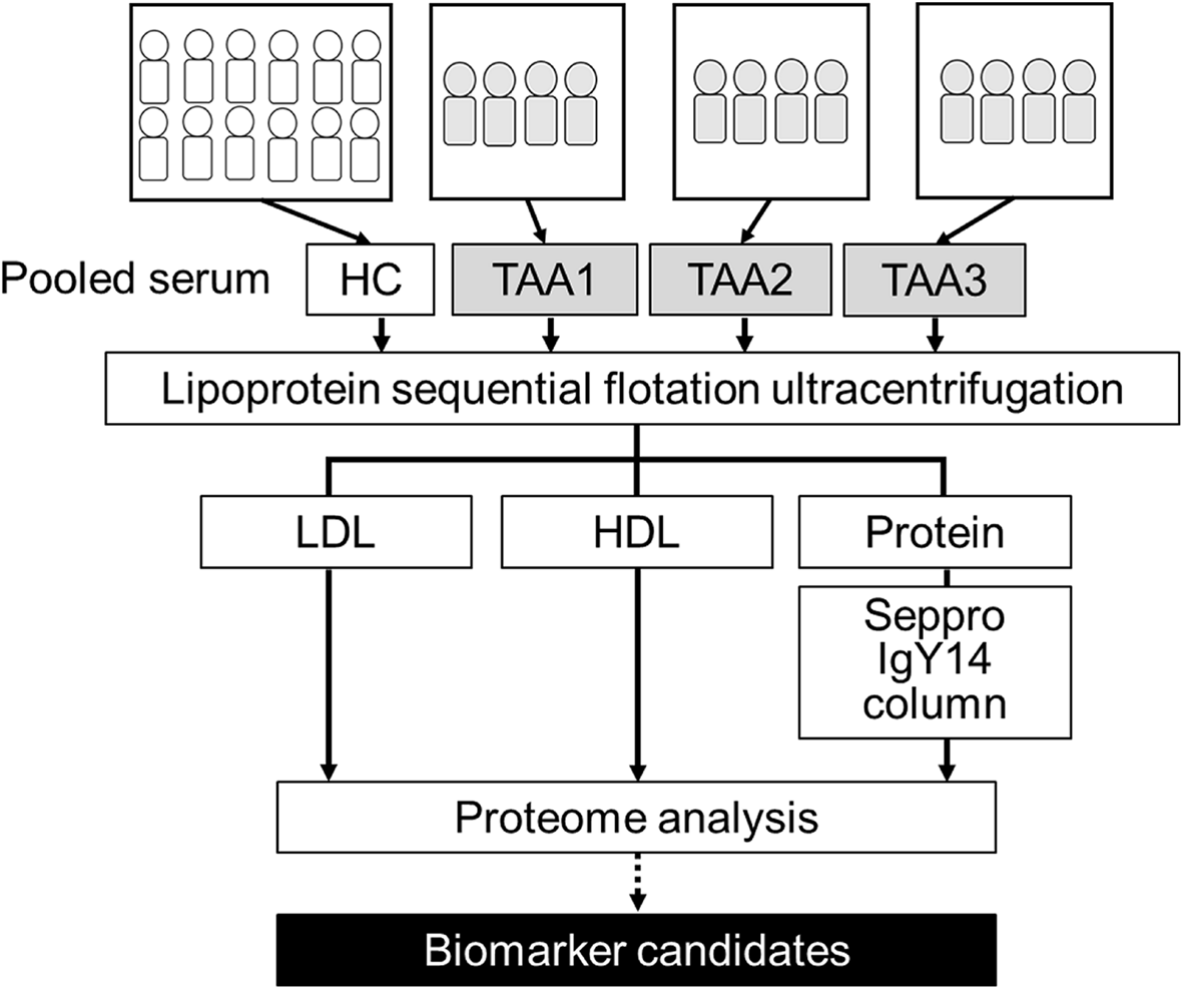
Preparation of LDL, HDL, and protein fractions from patients with TAA and HC subjects. Each pooled TAA serum sample was prepared with samples from 4 patients with TAA and pooled HC serum sample was prepared with samples from 12 HC subjects

To identify novel diagnostic biomarkers of AA measurable in blood samples, we performed proteome analysis of serum samples collected from patients with atherosclerotic TAA and HC subjects. Figure 1 shows a flowchart for the preparation of fractionated serum samples subjected to proteome analysis. To reduce the disturbance of highly abundant serum proteins, we employed a lipoprotein SFUC method for sample preparation. Three pooled serum samples from each of the four patients with TAA (TAA1, TAA2, and TAA3) and one pooled sample from 12 HC subjects (HC) were fractionated into three SFUC fractions: LDL, HDL, and protein fractions. From the protein fractions, 14 high-abundance serum proteins were depleted using Seppro IgY14 columns. All fractions were reduced and alkylated, digested with lysyl endopeptidase and trypsin, and subjected to non-target proteome analysis. A total of 346 proteins were identified in at least one of the fractionated samples (Additional File 2).

### PFN1 and CFD as biomarker candidates for TAA

**Fig. 2:**
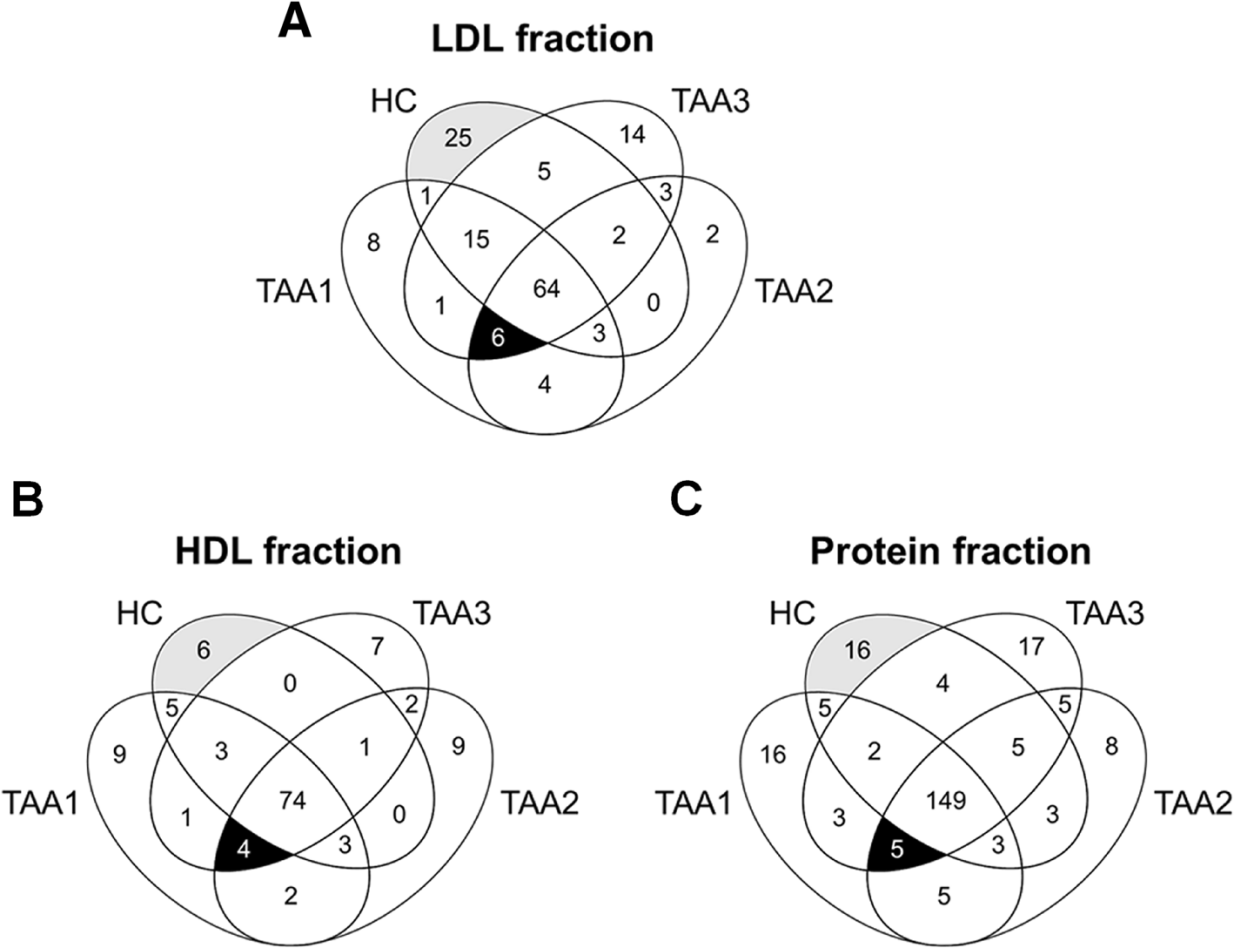
Identified proteins in LDL, HDL, and protein fractions of patients with TAA and HC subjects. The (**A**) LDL fraction, (**B**) HDL fraction, and (**C**) protein fraction prepared from three pooled serum samples of patients with TAA (TAA1, TAA2, and TAA3) and one pooled sample of HC subjects (HC) were subjected to proteome analysis, and the identified proteins in each sample were compared using Venn diagrams. The number of identified proteins was 153, 126, and 246 in the LDL, HDL, and protein fractions, respectively. In total, 346 independent proteins were identified in the serum samples of patients with TAA and HC subjects (Additional File 2). The black area indicates the number of proteins commonly detected in the three TAA samples but not in the HC sample of each SFUC fraction (TAA-only group), whereas the grey area indicates that detected in the HC sample but not in the three TAA samples of each SFUC fraction (HC-only group) (Additional Files 3–5)

The number of identified proteins in the LDL fractions (Fig. 2A), HDL fractions (Fig. 2B), and protein fractions (Fig. 2C) of TAA1, TAA2, TAA3, and HC serum samples are shown as Venn diagrams. We first selected the proteins commonly identified in three TAA samples, but not in the HC sample of each SFUC fraction (TAA-only group), or those detected in the HC sample, but not in the three TAA samples of each SFUC fraction (HC-only group), whose levels were considered to increase when compared with those of their counterpart fractions. From the LDL fractions of TAA and HC serum samples, 153 proteins were identified, of which 6 were in the TAA-only group, whereas 25 were in the HC-only group (Additional File 3). In the HDL fractions, 126 proteins were identified; 4 were detected in the TAA-only group and 6 in the HC-only group (Additional File 4). In the protein fraction, 246 proteins were identified; 5 and 16 proteins were identified in the TAA- and the HC-only groups, respectively (Additional File 5). Among all proteins listed in Additional Files 3–5, PFN1 was commonly identified in the three SFUC fractions of the HC-only group, but not in those of the three TAA samples, as summarised in Table 1. In contrast, CFD was identified in every SFUC fraction of the TAA-only group but not in that of the HC sample. Among the other proteins listed in Additional Files 3–5, immunoglobulin lambda variable 8–61 was identified in two of the three SFUC fractions of the HC-only group but not in any SFUC fraction of the TAA samples. The remaining proteins were identified once in one of the three SFUC fractions of the HC-only or TAA-only group. Taken together, PFN1 and CFD show highly contrasting identification profiles between TAA and HC serum samples, indicating a high probability that these protein levels increased or decreased in the serum samples of patients with TAA in comparison with those of the HC subjects. Thus, we considered PFN1 and CFD as the most probable biomarker candidates and performed confirmation and validation experiments to determine their potential as biomarkers of TAA.

**Table 1:** Summary of mass spectrometric identification of biomarker candidates in the LDL, HDL, and protein fractions

Detection group	Fraction	Gene symbol
HC-only^a^	LDL	*ATP9A*, *CRK*, *SPECC1L*, *FN1*, *GIGYF2*, *HRG*, *IGHV3-15, SERPING1*, Immunoglobulin epsilon heavy chain^c^, *IGHG3*, *ITIH1*, *KRT6C*, *IGKV3D*-*20*, I*GLV1*-*47*, *IGLV8*-*61*, *MARK4*, *MYO10*, *URB1*, ***PFN1***, *PSAP*, *SCN2A*, *SDPR*, *TAGLN2*, *TMEM198*, *TSHZ3*
	HDL	*IGFALS*, *APOH*, *IGKV2D-26*, ***PFN1***, *SHBG*,* ZNF587B*
	Protein	*APOB*, *BAZ1A*, *CDH5*, *COMP*, *GZMM*, *HRNR*, *IFT46*, *IGLV8-61*, *MSH3*, *MCAM*, *NCAM1*, *NEFL*, *PRDX1*, ***PFN1***, *PTPRG*,* SPP2*
TAA-only^b^	LDL	*APMAP*, *B2M*, ***CFD***, *C4A*, *RYR2*,* SAA1*
	HDL	*CFB*, ***CFD***, *LUM*,* SLX4*
	Protein	*ORM2*, ***CFD***, *CRP*, *FBLN1*, Immunoglobulin alpha-2 heavy chain^c^

Bold font indicates commonly identified genes in the three SFUC fractions of either the HC-only or TAA-only group

^a^Genes commonly detected in the three SFUC fractions of the HC-only group, but not in those of the three TAA serum samples (Additional Files 3, 4﻿ and 5)

^b^Genes commonly detected in the three SFUC fractions of the TAA-only group but not in those of the HC serum sample (Additional Files 3–5)

^c^Biomarker candidates are shown by protein names as they were not assigned to specific genes

### Alterations of biomarker candidates in unfractionated serum samples of patients with TAA and HC subjects

**Fig. 3:**
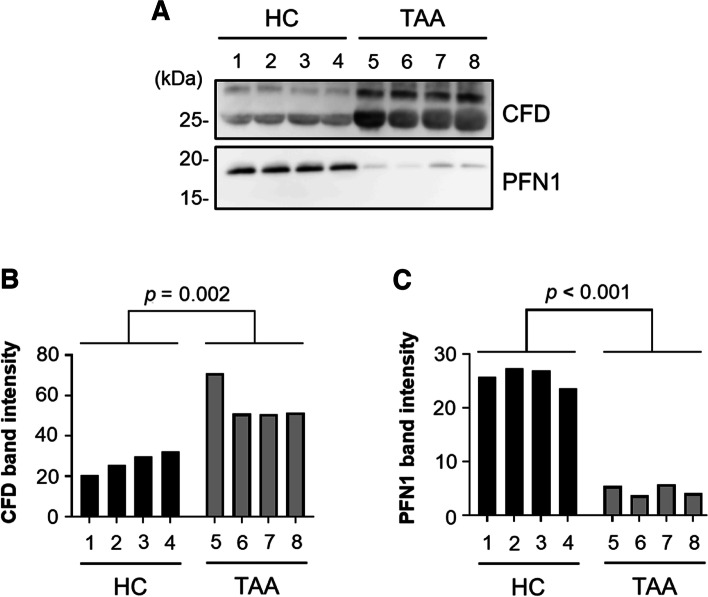
PFN1 and CFD levels in unfractionated serum samples of patients with TAA and HC subjects. **A** Western blot analysis of PFN1 and CFD in four individual serum samples without pre-treatment from patients with TAA and those from HC subjects. **B**, **C** The histograms indicate quantitated band intensity data of (**B**) PFN1 and (**C**) CFD in the serum samples of patients with TAA and HC subjects. HC subjects, No. 1–4; TAA patients, No. 5–8. Significant differences were observed between the TAA and HC groups. Images of entire western blots of PFN1 and CFD are shown in Additional File 8

To confirm the alterations in PFN1 and CFD concentrations between patients with TAA and the HC subjects, we performed western blot analysis of the unfractionated serum samples using antibodies specific to these proteins. The diluted serum samples from four patients and four controls were individually separated using SDS-PAGE, blotted on membranes, and detected with anti-PFN1 and anti-CFD antibodies (Fig. 3). The band intensity of PFN1 was attenuated in the serum samples of four patients with TAA, whereas the band intensity of CFD increased in the serum samples from all patients with TAA. These alterations in the unfractionated serum samples were consistent with those expected from the proteome analysis data of patients with TAA and the HC subjects.

### Serum concentrations of PFN1 and CFD and their ROC analysis in the discovery study

Using ELISA kits, we measured PFN1 and CFD concentrations in the serum samples of all patients with TAA (*n* = 29), patients with AAA (*n* = 41), and HC subjects (*n* = 44) in the discovery study. The characteristics of these patients and HC subjects are summarised in Additional File 1. These blood samples were collected from patients whose TAA tissues were previously used for identification of NPC2 and IGFBP7 [15]. Compared with the HC subjects, both patients with TAA and AAA were older with lower levels of HDL cholesterol (HDL-C) and higher levels of triglycerides, uric acid, and CRP, in addition to a higher body mass index (BMI) in patients with AAA.

**Fig. 4:**
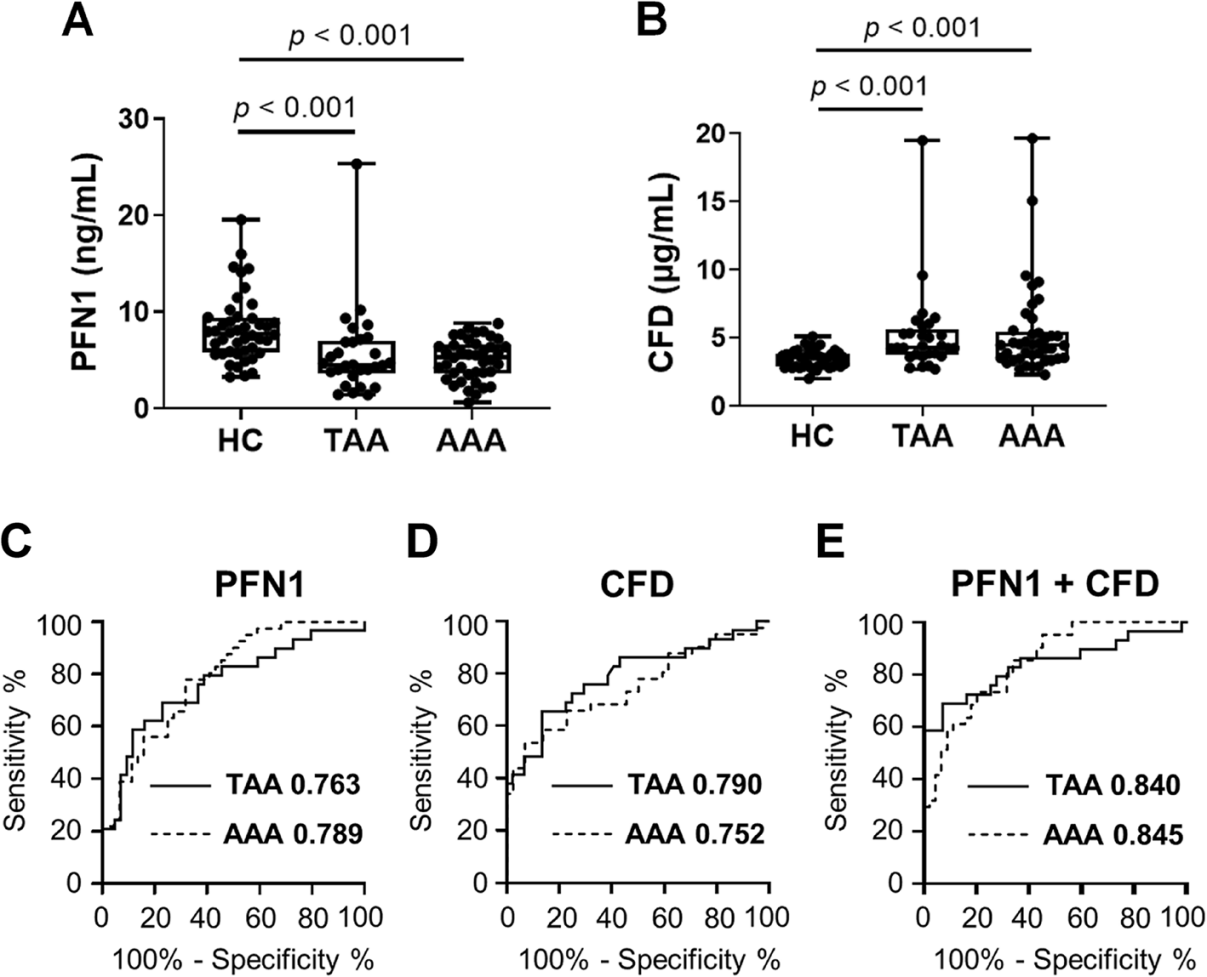
Serum PFN1 and CFD concentrations in patients with TAA and AAA in the discovery study. **A**, **B** In the discovery study, the concentrations of (**A**) PFN1 and (**B**) CFD in serum samples from 44 HC subjects, 41 patients with AAA, and 29 patients with TAA were measured using commercially available ELISA kits specific for each protein. Data are expressed as box-and-whisker plots. The centre line indicates the median value, the boxes indicate the interquartile range, and the whiskers indicate the upper or lower quartile range. Significant differences were observed between the TAA and HC groups and between the AAA and HC groups. **C**–**E** ROC analysis of (**C**) PFN1, (**D**) CFD, and (**E**) their combination (PFN1 + CFD) between the TAA and HC groups (solid line) or between the AAA and HC groups (dashed line). The AUCs of PFN1, CFD, and their combination (PFN1 + CFD) to discriminate the TAA or AAA group from HC group are shown in each figure following the disease names

The serum concentration of PFN1 was significantly lower in patients with TAA and AAA (TAA: *p* < 0.001 and AAA: *p* < 0.001) than in the HC subjects (Fig. 4A). In contrast, the serum concentration of CFD was significantly higher in patients with TAA and AAA (TAA: *p* < 0.001 and AAA: *p* < 0.001) than in the HC subjects (Fig. 4B). We performed ROC analysis of PFN1 and CFD between patients with TAA and HC subjects, or between patients with AAA and HC subjects. The AUC of PFN1 and CFD was 0.763 and 0.790 between patients with TAA and HC subjects, respectively (Fig. 4C and D). The AUC of PFN1 and CFD between patients with AAA and HC subjects was 0.789 and 0.752, respectively (Fig. 4C and D). In addition, we evaluated the combined AUC for these two protein biomarker candidates, which was 0.840 and 0.845 in patients TAA and AAA, respectively (Fig. 4E). Taken together, we concluded that PFN1 and CFD are novel biomarkers of TAA and AAA.

### Serum concentrations of PFN1 and CFD and their diagnostic capability in the validation study

Table 2 lists the characteristics of patients with atherosclerotic TAA (*n* = 24) and AAA (*n* = 39) enrolled in the validation study. As the blood samples from the HC subjects were not available in the NCVC Biobank, patients with IA (*n* = 21) without a clinical history of AA and its related diseases, hypertension, or diabetes were used as control subjects. The patients with TAA and AAA were older than the control patients with IA and generally had a higher BMI and triglyceride level and lower HDL-C levels. There were no significant differences in patient characteristics between the TAA and AAA groups, except for the serum level of triglycerides. As the aortic diameters of patients with TAA and AAA in the validation study (49.0 ± 1.3 nm and 44.6 ± 1.3 mm) were smaller than those in the discovery study (57.0 ± 1.3 nm and 52.6 ± 1.3 mm, *p* < 0.0001 in both comparisons), average progression stages of patients with AA were milder in the validation study.

**Table 2:** Characteristics of patients with TAA and AAA, and control patients with IA in the validation study

Parameter	TAA		AAA		control IA	
	**n**	**mean ± SEM**	**n**	**mean ± SEM**	**n**	**mean ± SEM**
Age (yo)	24	67.6 ± 2.3*	39	71.1 ± 1.1*	21	47.1 ± 1.9
Sex, male (n)	16	-	33	-	19	-
Sex, female (n)	8	-	6	-	2	-
Aortic diameter (mm)	22^a^	49.0 ± 1.3	34^†^	44.6 ± 1.3	-	-
BMI (kg/m^2^)	24	24.0 ± 0.5*	39	23.8 ± 0.5*	21	21.5 ± 0.7
Total cholesterol (mg/dL)	23^a^	180 ± 6	38^†^	187 ± 5	20^†^	194 ± 7
LDL-C (mg/dL)	22^a^	99 ± 6	37^†^	106 ± 5	19^†^	107 ± 5
HDL-C (mg/dL)	22^a^	52 ± 3*	38^†^	49 ± 2*	20^†^	66 ± 3
Triglycerides (mg/dL)	22^a^	142 ± 13*	37^†^	181 ± 20*	20^†^	90 ± 8
Uric acid (mg/dL)	23^a^	5.5 ± 0.3	38^†^	5.9 ± 0.2*	20^†^	5.0 ± 0.3
CRP (mg/dL)	24	0.21 ± 0.13	37^†^	0.17 ± 0.03	19^†^	0.13 ± 0.07

*LDL-C* low-density lipoprotein cholesterol, *yo* years old

Data are presented as mean ± SEM. *p < 0.05 when compared with control patients with IA

^a^The indicated number was less than the summation of the number of male and female patients in the TAA, AAA, and control IA groups owing to the unavailability of NCVC Biobank clinical data

**Fig. 5:**
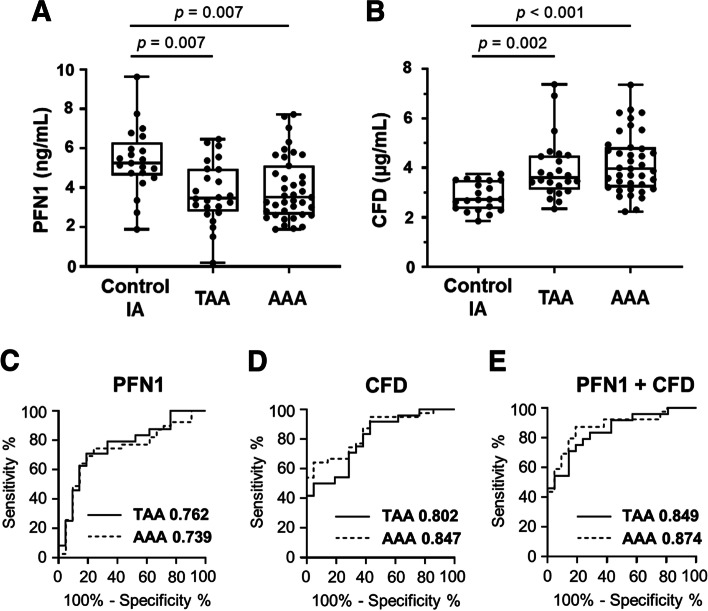
Serum PFN1 and CFD concentrations in patients with TAA and AAA in the validation study. **A**, **B** In the validation study, the concentrations of (**A**) PFN1 and (**B**) CFD in serum samples from 21 control patients with IA, 24 patients with TAA, and 39 patients with AAA were measured using commercially available ELISA kits specific for each protein. Data are expressed as box-and-whisker plots. The centre line indicates the median value, the boxes indicate the interquartile range, and the whiskers indicate the upper or lower quartile range. Significant differences were observed between the TAA and control IA groups and between the AAA and control IA groups. **C**–**E** ROC analysis of (**C**) PFN1, (**D**) CFD, and (**E**) their combination (PFN1 + CFD) between the TAA and control IA groups (solid line) or between the AAA and control IA groups (dashed line). The AUCs of PFN1, CFD, and their combination (PFN1 + CFD) to discriminate the TAA or AAA group from the control IA group are shown in each figure following the disease names

The PFN1 and CFD concentrations measured in the validation study were significantly altered in patients with TAA (*p* = 0.007 and *p* = 0.002, respectively) and AAA (*p* = 0.007 and *p* < 0.001, respectively) when compared with those in control patients with IA (Fig. 5A and B), verifying consistency with our data in the discovery study (Fig. 4A and B). There were no differences in the concentrations of the two biomarkers between patients with TAA and AAA. These results indicate that the two newly identified biomarkers are useful for the detection of patients with AA. The ROC analyses of these biomarkers were performed between patients with TAA or AAA and control patients with IA registered in the NCVC Biobank (Fig. 5C and D). The AUCs obtained from the ROC analysis of PFN1 slightly decreased in patients with AAA of the validation study, whereas those of CFD in the validation study increased to 0.802 and 0.847 in patients with TAA and AAA, respectively, when compared with the discovery study. We also evaluated the combined effects of PFN1 and CFD (Fig. 5E). After combining them, the AUC values of the biomarkers increased to 0.849 and 0.874 between patients with TAA and control patients with IA and between patients with AAA and control patients with IA, respectively.

### Serum concentrations of PFN1 and CFD in patients with AD

We measured the serum concentrations of PFN1 and CFD in patients with AD registered in the NCVC Biobank (*n* = 12, Additional File 6) although the number of patients was small. The serum concentrations of PFN1 and CFD showed significant differences between patients with AD and control patients with IA (PFN1: *p* < 0.001 and CFD: *p* = 0.009) (Additional File 7A and B). Particularly, PFN1 showed a higher diagnostic power (AUC: 0.849) than CFD (AUC: 0.774) through ROC analysis between patients with AD and control patients with IA (Additional File 7C). These results suggest that PFN1 is also effective in detecting patients with AD.

## Discussion

To develop a precise and accurate diagnostic system for detecting patients with AA, it is essential to identify more biomarkers reflecting the different aspects of pathogenesis and pathophysiology of AA. Thereafter, the most suitable biomarker, or a biomarker panel, should be selected using a clinical validation study. Our previous study data indicated a low efficiency of finding useful diagnostic biomarkers using the tissue proteome analysis method [15], and the direct identification of biomarkers from the blood samples remained challenging. Nevertheless, in the present study, we preformed proteome analysis of serum samples from patients with atherosclerotic TAA and HC subjects after pre-treatment using lipoprotein SFUC methods. The fractionation of serum samples into three SFUC fractions can efficiently reduce abundant serum proteins, and the proteome analysis of the LDL and HDL fractions can increase the probability of identifying new biomarkers involved in aneurysm and atherosclerosis. This approach was supported by the following evidence: (i) several proteins that play major roles in the development of atherosclerosis have been found in our previous proteome analysis of the eluates from LDL-apheresis adsorption columns [23] and (ii) a new biomarker for AAA has been identified using proteome analysis of the HDL fraction prepared from the plasma of patients with AAA [24]. By comparing the proteome analysis data of the three SFUC fractions obtained from patients with atherosclerotic TAA and HC subjects, we identified and selected two diagnostic biomarker candidates, PFN1 and CFD.

After acquiring the proteome data, we first attempted to sort out biomarker candidates from the proteins listed in Additional Files 3–5 that probably altered between the respective SFUC fractions of the TAA and HC groups (Fig. 2). As it was difficult to quantitatively compare protein levels after fractionation, even if corrected by recovery yields of proteins, we searched for proteins that were uniformly observed in all three SFUC fractions of either the HC group or the TAA group (Additional Files 3–5). PFN1 was commonly detected in the three SFUC fractions of the HC-only group, whereas CFD was commonly identified in those of the TAA-only group (Table 1). Thus, we selected PFN1 and CFD as primary candidates and performed western blot analyses with unfractionated serum samples to avoid pre-treatments. Significant changes in the band intensity of PFN1 and CFD were observed in the untreated serum samples between the TAA and HC groups (Fig. 3, Additional File 8). These two proteins were selected as the most probable biomarker candidates for identifying patients with TAA using serum samples.

As expected from the proteome and western blot analyses, the serum PFN1 concentration decreased, whereas the CFD concentration increased in the TAA and AAA groups when compared with those in the HC group (Fig. 4A and B). This reflects the technical merits of the biomarker search starting from the blood samples.

In the ROC analysis of PFN1 and CFD between the TAA and HC groups or between the AAA and HC groups in the discovery study, the AUCs of PFN1 and CFD were greater than 0.75 in patients with TAA and AAA. When both PFN1 and CFD were used together, the AUCs increased to over 0.84. The diagnostic power that discriminates patients with TAA or AAA from HC subjects was less than that of our previously identified NPC2 and IGFBP7 for TAA but close to that for AAA. We concluded that PFN1 and CFD are novel biomarkers for TAA and AAA, and their diagnostic utility should be validated in separate clinical samples.

PFN1 is a small actin-binding protein composed of 139 amino acid residues, mutations of which cause amyotrophic lateral sclerosis [25, 26]. Several studies have reported the functional contribution of PFN1 to vascular diseases [27, 28]. In a proteome analysis of TAA and AAA tissues, the expression of PFN1 decreased in calcified tissues [29]. In mice overexpressing PFN1 in blood vessels, hypertension was induced along with vascular wall thickening [30]. These findings suggest that PFN1 is not merely a cytoskeleton-forming protein with cytoplasmic actin but is rather functionally associated with vascular tissue organisation and maintenance, thus participating in the onset and progression of vascular diseases. The decrease in the concentration of PFN1 in the circulating blood of patients with TAA and AAA may be caused by the reduction in PFN1 level in the vascular tissues via remodelling and loss of aortic tissue integrity.

CFD is a circulating serine protease of 228 residues, which is a member of the alternative pathway of complement activation [31]. As CFD is mainly expressed and secreted from adipose tissues, this protein is recognised as an adipokine called ‘adipsin’ and presumed to circulate in a uniform molecular form [32]. In practice, the CFD concentration in the blood has been shown to be altered in metabolic diseases [33]. In cardiovascular diseases, circulating CFD concentration is associated with the progression of coronary heart diseases and could be used as a prognostic biomarker of this disease [34, 35]. Plasma CFD concentration has also been reported to be increased in patients with heart failure and systemic sclerosis [36]. Here, we provided the first evidence that serum CFD concentration is a diagnostic biomarker for TAA and AAA. It is likely that chronic inflammatory reactions accompanying the activation of alternative pathways of the complement system may contribute to the progression of aortic wall remodelling in atherosclerotic AA [37].

To evaluate the diagnostic capability of PFN1 and CFD, we performed a validation study using NCVC Biobank-registered patients with atherosclerotic AA. The number of patients with TAA and AAA in the validation study was not large and these patients generally showed milder clinical manifestations than those in the discovery study (Table 2). Nevertheless, the serum concentrations of PFN1 and CFD showed significant differences between the TAA and control IA groups and between the AAA and control IA groups as observed in the discovery study (Fig. 5A and B). There were no differences in the concentrations of the two biomarkers between the TAA and AAA groups in the validation and discovery studies. The AUC of CFD was higher in the validation study than in the discovery study, whereas that of PFN1 was almost comparable in both studies. Overall, PFN1 and CFD were confirmed to have reproducible diagnostic capabilities for discriminating patients with TAA and AAA from control patients with IA in the validation study.

In patients with TAA and AAA, the combination of PFN1 and CFD had high AUC values (0.840 and 0.845, respectively) in the discovery study (Fig. 4E). In the validation study, the combined AUC values of these biomarkers slightly increased in patients with TAA (0.849) and AAA (0.874) (Fig. 5E). These two potential biomarkers showed sufficient performance in detecting AA in both discovery and validation studies, warranting further assessment in a large-scale clinical study.

We also measured the serum concentrations of the two new biomarkers in patients with AD registered in the NCVC Biobank for their characterisation and application to related diseases (Additional File 6). Although the number of patients was small (*n* = 12), the serum concentrations of CFD and PFN1 were significantly different between the AD and control IA groups, and a higher AUC value (0.849) was observed for PFN1 (Additional File 7). As AD is a critical risk factor for aortic rupture and shares several pathogenic mechanisms with AA, the diagnostic performance of these biomarkers for detecting patients with AD may be worth exploring.

### Limitations of the study

This study has several limitations. First, there were significant differences in age between patients with TAA/AAA and HC subjects/control patients with IA in both discovery and validation studies. Second, the number of samples available for the validation study was not large, and IA patients without AA or related diseases were used as the control subjects. Third, the discovery study started from a limited number of pooled serum samples, and no quantitative comparison in the proteome analysis was performed to create a list of biomarker candidates. Therefore, the data presented in this study must be interpreted considering these limitations.

In conclusion, we discovered two novel biomarkers for AA, namely PFN1 and CFD, from protein data acquired using serum proteome analysis of patients with atherosclerotic TAA. These biomarkers showed sufficient capability to discriminate patients with TAA and AAA from HC in the discovery study and were confirmed to have satisfactory diagnostic power for detecting patients with AA in the validation study. Moreover, the diagnostic performance of these biomarkers was augmented by their combination. Based on these findings, we consider that the two biomarkers have high application potential in clinical diagnosis and the development of biomarker panels for AA diagnosis.

### Supplementary Information

**Additional file 1.** Characteristics of patients with TAA and AAA, and HC subjects enrolled in the discovery study.

**Additional file 2.** List of proteins identified in the LDL, HDL, and protein fractions

**Additional file 3.** Mass spectrometric identification of biomarker candidates in the LDL fraction.

**Additional file 4.** Mass spectrometric identification of biomarker candidates in the HDL fraction.

**Additional file 5.** Mass spectrometric identification of biomarker candidates in the protein fraction.

**Additional file 6.** Characteristics of patients with AD enrolled from the NCVC Biobank-registered patients (validation study).

**Additional file 7.** Serum concentrations of PFN1 and CFD in patients with AD and control patients with IA and their ROC analysis.

**Additional file 8.** Images of entire western blots of PFN1 and CFD in Figure 3A.

## Data Availability

The proteome analysis datasets generated and/or analysed during the current study are available in the jPOST repository [38]; the accession numbers are PXD033774 for ProteomeXchange and JPST001581 for jPOST. All other data generated and/or analysed during this study are included in this published article and its Additional Files, except for individual data including clinical and health examination data, which cannot be made publicly available per the approved protocols.

## Abbreviations

AA: Aortic aneurysm
AAA: Abdominal aortic aneurysm
AD: Aortic dissection
AUC: Area under the curve
BMI: Body mass index
CFD: Complement factor C
CRP: C-reactive protein
ECC: Eiken Chemical Co., Ltd.
ELISA: Enzyme-linked immunosorbent assays
HC: Healthy control
HDL: High-density lipoprotein
HDL-C: High-density lipoprotein cholesterol
IA: Inherited arrhythmia
LDL: Low-density and ultralow-density lipoprotein
LDL-C: Low-density lipoprotein cholesterol
NCVC: National Cerebral and Cardiovascular Center
NPC2: Niemann-Pick disease type C2 protein
IGFBP7: Insulin-like growth factor-binding protein 7
MS: Mass spectrometric
LC–MS/MS: Liquid chromatography-tandem mass spectrometric
PFN1: Profilin 1
ROC: Receiver operating characteristic
SFUC: Sequential flotation ultracentrifugation
TAA: Thoracic aortic aneurysm
yo: Years old
