# Supplementary material for: Serum proteomic identification and validation of two novel atherosclerotic aortic aneurysm biomarkers, profilin 1 and complement factor D
Source: Proteome Sci. 2023 Aug 5;21:11. doi: 10.1186/s12953-023-00212-x (PMC10403969; doi:10.1186/s12953-023-00212-x)
Supplement: Supplementary file 1 — Additional file 1. Characteristics of patients with TAA and AAA, and HC subjects enrolled in the discovery study. [file 12953_2023_212_MOESM1_ESM.pdf]

Additional File 1: Characteristics of patients with TAA and AAA, and HC subjects enrolled in the discovery study.

Parameter	TAA	AAA	HC
Number total (n)	29	41	44
Age (yo)	71.1 ± 1.4*	68.5 ± 1.3*	47.4 ± 1.7
Male (n)	27	37	24
Female (n)	2	4	20
Aortic diameter (mm)	57.0 ± 1.3	52.6 ± 1.3	-
Smokers (n)	24	35	19
BMI (kg/m^2)†	23.3 ± 0.6	24.0 ± 0.5*	22.1 ± 0.4
Systolic blood pressure (mmHg)	122 ± 3	116 ± 2	116 ± 2
Diastolic blood pressure (mmHg)	70 ± 2	63 ± 2	69 ± 2
Total cholesterol (mg/dL)	172 ± 6	180 ± 5	187 ± 4
LDL-C (mg/dL)	100 ± 5	107 ± 5	102 ± 3
HDL-C (mg/dL)	51 ± 2*	46 ± 2*	74 ± 2
Triglycerides (mg/dL)	121 ± 14*	149 ± 12*	64 ± 4
Uric acid (mg/dL)	6.1 ± 0.2*	6.1 ± 0.3*	5.0 ± 0.2
CRP (mg/dL)	0.16 ± 0.04*	0.39 ± 0.12*	0.06 ± 0.03

Data are presented as mean ± SEM. *$p < 0.05$ compared with HC. †One body weight value of the HC group was unavailable and hence excluded from the data analysis of BMI. AAA, abdominal aortic aneurysm; BMI, body mass index; CRP, C-reactive protein; HC, healthy control; HDL-C, high-density lipoprotein cholesterol; LDL-C, low-density lipoprotein cholesterol; TAA, thoracic aortic aneurysm; yo, years old.
