# Supplementary material for: Serum proteomic identification and validation of two novel atherosclerotic aortic aneurysm biomarkers, profilin 1 and complement factor D
Source: Proteome Sci. 2023 Aug 5;21:11. doi: 10.1186/s12953-023-00212-x (PMC10403969; doi:10.1186/s12953-023-00212-x)
Supplement: Supplementary file 3 — Additional file 3. Mass spectrometric identification of biomarker candidates in the LDL fraction. [file 12953_2023_212_MOESM3_ESM.pdf]

Additional File 3: Mass spectrometric identification of biomarker candidates in the LDL fraction.

Protein	Gene name	HC	TAA1	TAA2	TAA3
Probable phospholipid-transporting ATPase IIA	ATP9A	+	-	-	-
Adapter molecule crk	CRK	+	-	-	-
Cytospin-A	SPECC1L	+	-	-	-
Fibronectin	FN1	+	-	-	-
GRB10-interacting GYF protein 2	GIGYF2	+	-	-	-
Histidine-rich glycoprotein	HRG	+	-	-	-
Immunoglobulin heavy variable 3-15	IGHV3-15	+	-	-	-
Plasma protease C1 inhibitor	SERPING1	+	-	-	-
Immunoglobulin epsilon heavy chain	N/A	+	-	-	-
Immunoglobulin heavy constant gamma 3	IGHG3	+	-	-	-
Inter-alpha-trypsin inhibitor heavy chain H1	ITIH1	+	-	-	-
Keratin, type II cytoskeletal 6C	KRT6C	+	-	-	-
Immunoglobulin kappa variable 3D-20	IGKV3D-20	+	-	-	-
Immunoglobulin lambda variable 1-47	IGLV1-47	+	-	-	-
Immunoglobulin lambda variable 8-61	IGLV8-61	+	-	-	-
MAP/microtubule affinity-regulating kinase 4	MARK4	+	-	-	-
Unconventional myosin-X	MYO10	+	-	-	-
Nucleolar pre-ribosomal-associated protein 1	URB1	+	-	-	-
Profilin-1	PFN1	+	-	-	-
Prosaposin	PSAP	+	-	-	-
Sodium channel protein type 2 subunit alpha	SCN2A	+	-	-	-
Serum deprivation-response protein	SDPR	+	-	-	-
Transgelin-2	TAGLN2	+	-	-	-
Transmembrane protein 198	TMEM198	+	-	-	-
Teashirt homolog 3	TSHZ3	+	-	-	-
Adipocyte plasma membrane-associated protein	APMAP	-	+	+	+
Beta-2-microglobulin	B2M	-	+	+	+
Complement factor D	CFD	-	+	+	+
Complement C4-A	C4A	-	+	+	+
Ryanodine receptor 2	RYR2	-	+	+	+
Serum amyloid A-1 protein	SAA1	-	+	+	+

+: Identified in this fraction, -: Not identified in this fraction, N/A: Not available.
HC, healthy control; TAA, thoracic aortic aneurysm.
