# Supplementary material for: Serum proteomic identification and validation of two novel atherosclerotic aortic aneurysm biomarkers, profilin 1 and complement factor D
Source: Proteome Sci. 2023 Aug 5;21:11. doi: 10.1186/s12953-023-00212-x (PMC10403969; doi:10.1186/s12953-023-00212-x)
Supplement: Supplementary file 4 — Additional file 4. Mass spectrometric identification of biomarker candidates in the HDL fraction. [file 12953_2023_212_MOESM4_ESM.pdf]

Additional File 4: Mass spectrometric identification of biomarker candidates in the HDL fraction.

Protein	Gene name	HC	TAA1	TAA2	TAA3
Insulin-like growth factor-binding protein complex acid labile subunit	IGFALS	+	-	-	-
Beta-2-glycoprotein 1	APOH	+	-	-	-
Immunoglobulin kappa variable 2D-26	IGKV2D-26	+	-	-	-
Profilin-1	PFN1	+	-	-	-
Sex hormone-binding globulin	SHBG	+	-	-	-
Zinc finger protein 587B	ZNF587B	+	-	-	-
Complement factor B	CFB	-	+	+	+
Complement factor D	CFD	-	+	+	+
Lumican	LUM	-	+	+	+
Structure-specific endonuclease subunit SLX4	SLX4	-	+	+	+

+: Identified in this fraction, -: Not identified in this fraction.
HC, healthy control; TAA, thoracic aortic aneurysm.
