# Supplementary material for: Serum proteomic identification and validation of two novel atherosclerotic aortic aneurysm biomarkers, profilin 1 and complement factor D
Source: Proteome Sci. 2023 Aug 5;21:11. doi: 10.1186/s12953-023-00212-x (PMC10403969; doi:10.1186/s12953-023-00212-x)
Supplement: Supplementary file 5 — Additional file 5. Mass spectrometric identification of biomarker candidates in the protein fraction. [file 12953_2023_212_MOESM5_ESM.pdf]

Additional File 5: Mass spectrometric identification of biomarker candidates in the protein fraction.

Protein	Gene name	HC	TAA1	TAA2	TAA3
Apolipoprotein B-100	APOB	+	-	-	-
Bromodomain adjacent to zinc finger domain protein 1A	BAZ1A	+	-	-	-
Cadherin-5	CDH5	+	-	-	-
Cartilage oligomeric matrix protein	COMP	+	-	-	-
Granzyme M	GZMM	+	-	-	-
Hornerin	HRNR	+	-	-	-
Intraflagellar transport protein 46 homolog	IFT46	+	-	-	-
Immunoglobulin lambda variable 8-61	IGLV8-61	+	-	-	-
DNA mismatch repair protein Msh3	MSH3	+	-	-	-
Cell surface glycoprotein MUC18	MCAM	+	-	-	-
Neural cell adhesion molecule 1	NCAM1	+	-	-	-
Neurofilament light polypeptide	NEFL	+	-	-	-
Peroxiredoxin-1	PRDX1	+	-	-	-
Profilin-1	PFN1	+	-	-	-
Receptor-type tyrosine-protein phosphatase gamma	PTPRG	+	-	-	-
Secreted phosphoprotein 24	SPP2	+	-	-	-
Alpha-1-acid glycoprotein 2	ORM2	-	+	+	+
Complement factor D	CFD	-	+	+	+
C-reactive protein	CRP	-	+	+	+
Fibulin-1	FBLN1	-	+	+	+
Immunoglobulin alpha-2 heavy chain	N/A	-	+	+	+

+: Identified in this fraction, -: Not identified in this fraction, N/A: Not available.
HC, healthy control; TAA, thoracic aortic aneurysm.
