# Supplementary material for: Serum proteomic identification and validation of two novel atherosclerotic aortic aneurysm biomarkers, profilin 1 and complement factor D
Source: Proteome Sci. 2023 Aug 5;21:11. doi: 10.1186/s12953-023-00212-x (PMC10403969; doi:10.1186/s12953-023-00212-x)
Supplement: Supplementary file 6 — Additional file 6. Characteristics of patients with AD enrolled from the NCVC Biobank-registered patients (validation study). [file 12953_2023_212_MOESM6_ESM.pdf]

Additional File 6: Characteristics of patients with AD enrolled from the NCVC Biobank-registered patients (validation study).

Parameter	AD	
	**n**	**mean ± SEM**
Age (yo)	12	63.3 ± 3.7
Sex, male (n)	8	-
Sex, female (n)	4	-
BMI (kg/m^2)	12	23.9 ± 1.3
Total cholesterol (mg/dL)	10†	181 ± 12
LDL-C (mg/dL)	10†	107 ± 12
HDL-C (mg/dL)	10†	48 ± 4
Triglycerides (mg/dL)	10†	152 ± 26
Uric acid (mg/dL)	12	6.2 ± 0.6
CRP (mg/dL)	12	1.00 ± 0.46

Data are presented as mean ± SEM. †The indicated number was less than that of patients in the AD group owing to the unavailability of the NCVC Biobank clinical data.
AD, aortic dissection; BMI, body mass index; CRP, C-reactive protein; HDL-C, high-density lipoprotein cholesterol; LDL-C, low-density lipoprotein cholesterol; NCVC, National Cerebral and Cardiovascular Center; yo, years old.
