# Supplementary material for: Serum proteomic identification and validation of two novel atherosclerotic aortic aneurysm biomarkers, profilin 1 and complement factor D
Source: Proteome Sci. 2023 Aug 5;21:11. doi: 10.1186/s12953-023-00212-x (PMC10403969; doi:10.1186/s12953-023-00212-x)
Supplement: Supplementary file 7 — Additional file 7. Serum concentrations of PFN1 and CFD in patients with AD and control patients with IA and their ROC analysis. [file 12953_2023_212_MOESM7_ESM.pdf]

Additional File 7: Serum concentrations of PFN1 and CFD in patients with AD and control patients with IA and their ROC analysis.

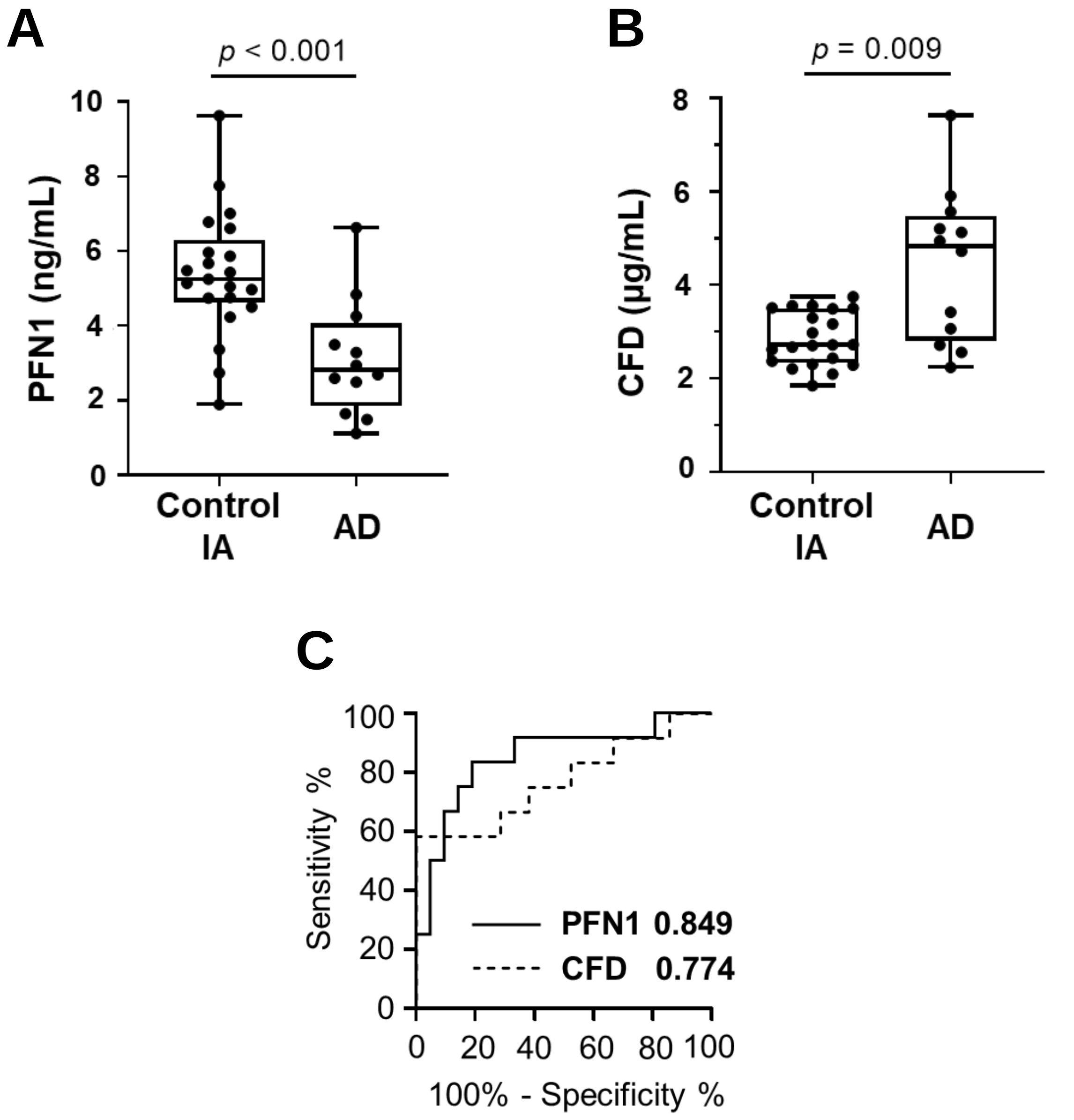

Serum concentrations of (A) PFN1 and (B) CFD in patients with AD and control patients with IA were measured using ELISA kits specific for each protein. Data are expressed as box-and-whisker plots. The centre line indicates the median value, the boxes indicate the interquartile range, and the whiskers indicate the upper or lower quartile range. Significant differences were observed in both PFN1 and CFD concentrations between the groups. (C) ROC curves of PFN1 and CFD between the AD and control IA groups are indicated by solid line and dashed line, respectively; the individual AUC values are shown following the biomarker names.

AD, aortic dissection; AUC, area under the curve; CFD, complement factor D; IA, inherited arrhythmia; PFN1, profilin 1; ROC, receiver operating characteristic.
