# Supplementary material for: Serum proteomic identification and validation of two novel atherosclerotic aortic aneurysm biomarkers, profilin 1 and complement factor D
Source: Proteome Sci. 2023 Aug 5;21:11. doi: 10.1186/s12953-023-00212-x (PMC10403969; doi:10.1186/s12953-023-00212-x)
Supplement: Supplementary file 8 — Additional file 8. Images of entire western blots of PFN1 and CFD in Figure 3A. [file 12953_2023_212_MOESM8_ESM.pdf]

Additional File 8: Images of entire western blots of PFN1 and CFD in Figure 3A.

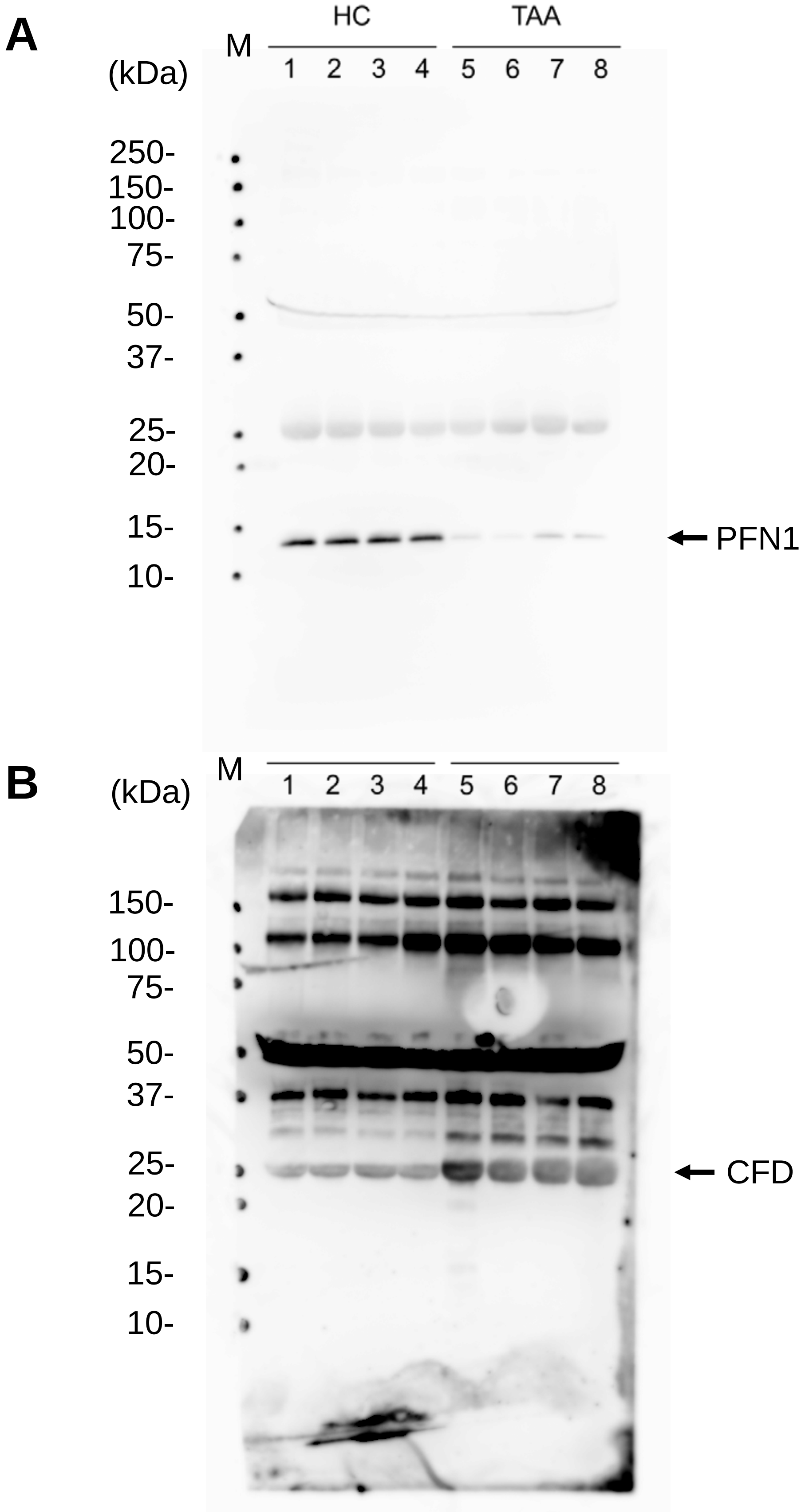

(A) PFN1 and (B) CFD levels in unfractionated serum samples from individual patients with TAA and HC subjects are shown. HC subjects, No. 1–4; TAA patients, No. 5–8. The predicted positions of PFN1 and CFD are indicated with arrows on the right, whereas the positions of molecular size markers (lane M) and their molecular masses in kDa are represented on the left.
CFD, complement factor D; HC, healthy control; PFN1, profilin 1; TAA, thoracic aortic aneurysm.
